# Dynamic measurements of speed and risk perception during driving: Evidence of speed misestimation from continuous ratings and video analysis

**DOI:** 10.1371/journal.pone.0291043

**Published:** 2023-09-01

**Authors:** Uijong Ju, Christian Wallraven

**Affiliations:** 1 Department of Information Display, Kyung Hee University, Seoul, South Korea; 2 Department of Brain and Cognitive Engineering, Korea University, Seoul, South Korea; 3 Department of Artificial Intelligence, Korea University, Seoul, South Korea; Texas A&M Transportation Institute, UNITED STATES

## Abstract

Investigating the factors underlying perceived speed and risk is crucial to ensure safe driving. However, existing studies on this topic usually measure speed and risk perception indirectly after a driving session, which makes it difficult to trace dynamic effects and time points of potential misestimates. To address this problem, we developed and validated a novel continuous method for dynamically measuring risk and speed perceptions. To study the factors affecting risk and speed perception, we presented participants with videos captured on the same racing track from the same point of view but with different drivers who varied in their speed and risk profiles. During the experiment, participants used a joystick to continuously rate the subjectively perceived risk of driving in the first block and the perceived speed in the second block. Our analysis of these dynamic ratings indicates that risk and speed estimates were decoupled, with curves resulting in decreased speeds but increased risk ratings. However, a close distance to the car in front increased both speed and risk. Based on actual and estimated speed data, we found that overtaking cars on curves resulted in participants overestimating their own speed, whereas an increase in the distance to the car in front on a straight course led to underestimations of their own speed. Our results showcase the usefulness of dynamic rating profiles for in-depth investigations into situations that could result in drivers misjudging speed or risk and will thus help the development of more intelligent, human-centered driving assistance systems.

## Introduction

In real-world situations, drivers must continuously process factors such as road traffic, weather, traffic signs, road characteristics, and in-vehicle information to adjust their driving behavior to the current situation. Errors in the perception of any of these factors can have disastrous consequences, and they have indeed been associated with increases in the likelihood of road crashes [1] and accidents in general [2, 3].

One of the most important factors in the continuous flow of traffic is the correct perception of speed, both in absolute terms—as it is not always possible to glance at the speedometer—and in relative terms, with respect to speed differences between one’s own and other cars. Perceived speed influences driving behaviors such as accelerating, braking, or changing lanes; perceiving speed accurately is thus crucial for safety [4], as well as the prevention of speeding violations [5]. However, drivers frequently overestimate or underestimate their speed. For example, after exiting a highway and entering an urban road, drivers typically underestimate how fast they are traveling [6]. Tunnel sidewall markings, on the other hand, often lead to drivers overestimating driving speed [7].

Previous studies have revealed several visual and environmental factors that critically influence speed perception. The overall and central fields of view, for instance, play an important role in speed perception [5], with decreases in the central field of view [8] leading to speed being underestimated. In-vehicle factors also affect the driver’s perception of their own speed; for example, vehicle noise reduces the variability of correct speed perception [9], whereas distraction induced by secondary tasks [10] and the absence of a speedometer both lead to speed underestimation [4]. Additionally, speed perception is influenced by external environmental factors, such as curves in the road. Research suggests that drivers often underestimate their speed on curves in both actual and simulated driving scenarios [11, 12].

Risk perception plays an important role in assessing driving speed, as it critically influences drivers’ attitudes toward road safety [13] and driving behavior [14]; impairments in risk perception can increase traffic accidents [2, 15]. An example of the latter effect are young drivers with low-risk perceptions who underestimate road hazards while overestimating their driving ability [16, 17], thereby causing accidents [18].

Critical factors for risk perception include personal characteristics such as age [19–21], driving experience [18, 20, 22], and sex [21, 23]. Environmental factors that impact risk perception include road characteristics, traffic conditions, and the behavior of vehicles ahead. An example of this impact is the change in perceived risk that occurs while driving on slopes [24]. Characteristics such as horizontal curves and narrow lanes can lead to drivers overestimating risk [25], as can dense traffic [26]. The perception of risk escalates when drivers are following other vehicles or when the following distance significantly decreases [27].

Several methods have been proposed to assess speed and risk perception and the role of both in driver behavior and traffic safety. Studies can be broadly categorized into experience-based self-reports involving questionnaires or structured interviews [2, 13, 14, 21], behavior-based experiments that include real-world [28, 29] or simulated [30–33] driving, and indirect experience-based research in which participants rate videos or images [19, 34–37]. However, the existing studies have critical limitations, as they have rarely been able to assess *dynamic* changes in the perceptions of speed or risk. Self-report-based surveys often simply provide an overall assessment of these factors rather than recording and analyzing dynamic changes in judgments over time. In addition, there is always a delay between the actual experience and a survey or post-experiment annotations, which raises questions about the reliability of such ratings. Finally, image- or video-based surveys often only use specific time points for annotations [19, 35–37] if they do not record only one average rating [34, 37].

This study addresses these limitations with a new experimental design based on continuous rather than post-stimulus measurements. A previous study showed that immediate verbal reports during an experiment showed better usability than a post-experiment questionnaire [38]. Similarly, by dynamically measuring changes in risk and speed perception during driving, we can accurately identify instances of misestimation compared to post-stimulus surveys or single time-points, which may introduce measurement errors from faulty recall. Therefore, in our study, we implemented dynamic assessment methods that can accurately identify such instances and may, in turn, contribute to accident prevention by mitigating errors in estimation. For this purpose, participants used a joystick to continuously rate perceived speed or risk as they watched footage of driving videos captured on the same racing track. These dynamic ratings allowed us to identify points at which speed and/or risk reached minima and maxima and assess correlations between these points among participants.

We thus tested the following four hypotheses to demonstrate the validity of the dynamic ratings: First, we expected subjective speed and risk to correlate with the actual speed of the car. Second, we assumed that the time points at which subjectively perceived speed and risk were at their minimum/maximum would be characterized by similar road conditions and situational factors. Third, we hypothesized that situational factors, perceived risk, and road characteristics are related. Fourth, we expected our correlational analysis of actual and estimated speeds to allow us to determine which driving situations lead to overestimations or underestimations of driving speed. Our findings demonstrate the practicality and validity of our dynamic rating approach in identifying scenarios in which drivers may miscalculate speed or risk; this approach could help in the development of driving assistance systems that ensure safety and reliability in situations where human error can occur.

## Materials and methods

### Dataset

We used a set of driving videos recorded on the Nürburgring race track in the town of Nürburg, Germany. All videos were downloaded from YouTube, and are available at the following URLs: https://www.youtube.com/watch?v=7BwDs3ja-WA (video 1), https://www.youtube.com/watch?v=JvqnUQpa2W4 (video 2), https://www.youtube.com/watch?v=kqzK9PEgVLA (video 3), https://www.youtube.com/watch?v=F3Ihb8RFV_g (video 4), https://www.youtube.com/watch?v=mj7dkeQcNPM (video 5), https://www.youtube.com/watch?v=QYV3yWshHLI (video 6), and https://www.youtube.com/watch?v=PlsjIj5O70w&t=314s (video 7). We selected videos with a 1920×1080 resolution to match the resolution of our monitor, decreased each video to 2 minutes of driving from the starting point of the racing track, and covered car logos and speedometers using a video visual effects application (Adobe After Effects; Adobe Inc., San Jose, CA, USA).

### Experimental design and procedure

**Fig 1:**
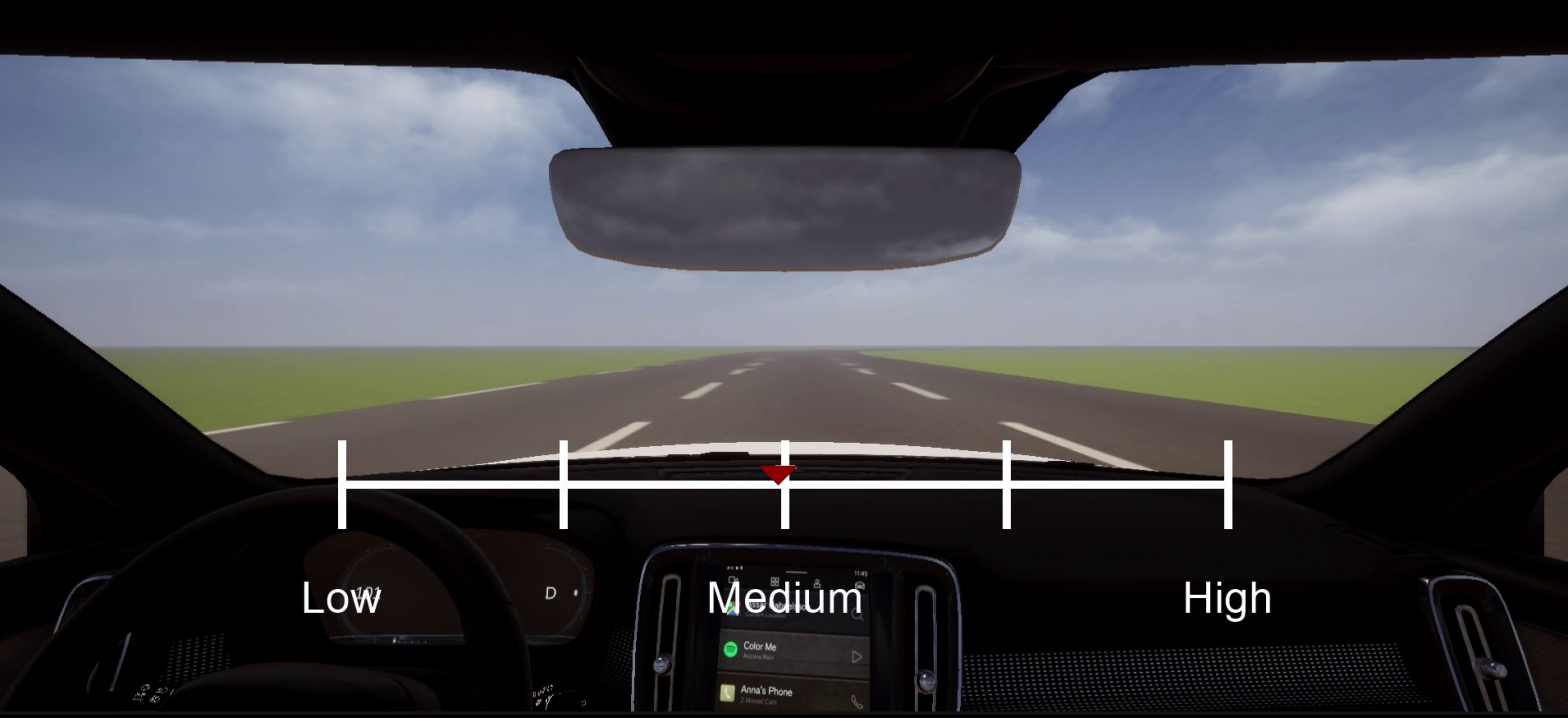
Screenshot of the experiment. Participants controlled the slider with a joystick, allowing for continuous ratings. Due to copyright issues, the figure has been replaced with a self-generated image using Unity 3D that is similar but not identical to the original image used in the study, and is therefore intended for illustrative purposes only.

Psychopy 3.0 (https://www.psychopy.org/) was used to display each video clip, overlaid with a sliding bar (see Fig 1), on a 27-inch LCD monitor (Samsung, Suwon, South Korea). A joystick (Action Joystick, Seoul, South Korea) interface enabled continuous control of the sliding bar.

Before the experiment, participants were informed about the tasks and the goal of the study and provided written consent. They were instructed to watch the videos and continuously rate the driving risk during the first block and the driving speed during the second block. In the first block, participants rated risk by choosing the most/least risky driving sequence; in the second block, they rated speed by choosing the slowest/fastest driving sequence. Risk perception may be influenced by order, as the speed perception task can provide feedback on the risk perception task; thus, rating speed before rating risk may affect subjective perceptions of risk [39]. However, order has relatively little influence on the rating of speed. Therefore, to avoid any effects on the rating of risk, we used a fixed order with a block-based design to evaluate risk before speed perception. Participants were informed that the videos were presented in a randomized order (with the order changing across blocks), and that after watching each video, they were to note their choices of the most and least risky, updating them accordingly as they progressed through the videos. At the end of the first block (risk), they selected the videos showing the least and most risky driving, and at the end of the second block (speed), they selected the videos showing the slowest and fastest driving. Furthermore, as the goal of the study was to investigate changes in dynamic perception, participants were instructed to only move the slider from neutral to any other position after the first 5 seconds of the video. The experimenter answered all of the participants’ questions before proceeding to the main experiment.

The main experiment began with 30 seconds of baseline instructions displayed on the screen (instructing participants to rate either risk or speed), and participants provided their ratings of the seven driving videos by continuously updating the slider. After each video, participants took a 10-second break to update their perceived risk/speed rankings of the videos they had already watched. After completing both blocks, participants were asked to confirm their selections of the most/least risky and slowest/fastest driving.

### Sample size and participants

To determine an adequate sample size, we first ran a pilot study with 15 licensed drivers (mean age = 26.07, SD = 3.53 years) in which the same videos were shown in a counterbalanced order. Participants were only asked to provide continuous ratings of car speed. This allowed us to assess the approximate correlation between actual and subjective speed, yielding an overall correlation value of r = 0.43, and negative correlations between actual speed and subjective risk (average r = −0.15) and subjective risk and subjective speed (average r = −0.40). Assuming a moderate to large correlation strength of r = 0.4 [40] for our study, the required sample size was determined using G* power [41]. Our two-tailed, point-biserial model correlation analysis, with values of r = 0.4, power = .9, alpha = .025 (Bonferroni-correction of N = 2 because we wanted to measure two correlations: speed and risk), yielded a required sample size of N = 68. We thus recruited 78 participants (34 female) from the student population of Korea University, ranging in age from 18 to 30 years (mean age = 23.5, SD = 3.27 years). Data collection took place between October 2020 and December 2020.

### Ethical statement

Our study was approved by the local ethics committee at Korea University (approval number: KUIRB-2020-0207-01). All methods and procedures were executed in compliance with the applicable guidelines and regulations, and the authors were not able to access information that could identify individual participants during or after data collection.

### Baseline analysis

First, actual speed, subjective risk, and subjective speed were extracted once every second, from the 0- to the 120-second time points, to investigate correlations between measurements. Second, average risk and speed were calculated by merging ratings across frames and dividing the value by the total number of frames. Third, we conducted correlational analyses using SPSS (Version 26.0, SPSS Inc., Chicago, IL, USA) to investigate associations between video content and participant ratings.

### Maximum and minimum perception points

We then extracted the maximum and minimum time points of actual speed, subjective risk, and subjective speed to characterize sequences that were perceived as dangerous versus safe or fast versus slow. To achieve this, we extracted 6-second clips from 3 seconds before to 3 seconds after each minimum and maximum time point, assessed them for road type (straight/curved), presence of cars in front (yes/no), and short following distance to the cars in front (yes/no). We then verified whether the actual speed matched the subjective speed (yes/no) at the minimum and maximum time points.

### Context-based driving risk, speed estimation

Next, we investigated the driving context to determine associations between overall risk and speed evaluations. To that end, we counted the total number of overtaking maneuvers, the total number of times the car in each video was overtaken by other vehicles, and the total number of times the distance to the car in front (if there was one) increased or decreased. Finally, we investigated associations between these measures and overall risk/speed evaluations (most/least risky and fastest/slowest driving), average dynamic risk, subjective speed, and actual speed.

### Dynamic analysis of speed over- and underestimation

We identified the time points in which speed overestimations and underestimations occurred in each video and compared these points with actual speed recordings and subjective speed estimations to determine what situations may lead to misestimations. We first normalized actual and subjective speeds by setting the maximum actual speed to 1, the minimum actual speed to 0, the maximum subjective speed rating to 1, and the minimum subjective speed rating to 0 for all videos. Next, we subtracted the normalized values from the actual and subjective speeds to identify the most overestimated and underestimated sequences in each video. We then extracted clips from 3 seconds before to 3 seconds after these extreme time points and analyzed the resulting 6-second sequences in terms of road characteristics (straight/curved), presence of cars in front (yes/no), and, if there was a car in front, the change in distance to that car within the extracted period (decreased/increased). We also analyzed the change in speed from the 3 seconds before to the 3 seconds after the extreme time point to identify road characteristics and driving situations that might lead to speed misestimations.

## Results

### Correlational analysis across behavioral ratings

To assess intercorrelations between the videos and ratings, we first averaged actual speed, subjective risk, and subjective speed across all time points, and assessed the actual and subjective maximum speeds across all seven videos (see Table 1). Rank ordering showed the highest correlation between maximum speed and actual speed (r = 0.93, p = .007) as well as high correlations between subjective speed and both maximum speed (r = 0.75, p = .063) and actual speed (r = 0.64, p = .139). However, the ranking for subjective risk showed comparatively lower correlations with the other measurements, including actual speed (r = 0.43, p = .353), maximum speed (r = 0.46, p = .302) and subjective speed (r = 0.43, p = .353).

**Table 1:** Basic characteristics: Actual and subjective driving speed, maximum speed, subjective risk.

	Actual speed (SD), km/h	Maximum speed, km/h	Subjective speed (SD)	Subjective risk (SD)
Video 1	161.21 (50.18)	272	3.54 (0.48)	3.14 (0.64)
Video 2	151.68 (46.77)	256	3.19 (0.52)	3.01 (0.61)
Video 3	136.65 (39.84)	221	2.89 (0.50)	3.11 (0.55)
Video 4	138.69 (39.26)	218	3.53 (0.52)	2.84 (0.74)
Video 5	147.00 (43.39)	245	3.20 (0.55)	3.19 (0.57)
Video 6	151.57 (43.79)	242	3.15 (0.50)	2.83 (0.67)
Video 7	114.18 (27.78)	171	3.06 (0.49)	2.62 (0.66)

**Fig 2:**
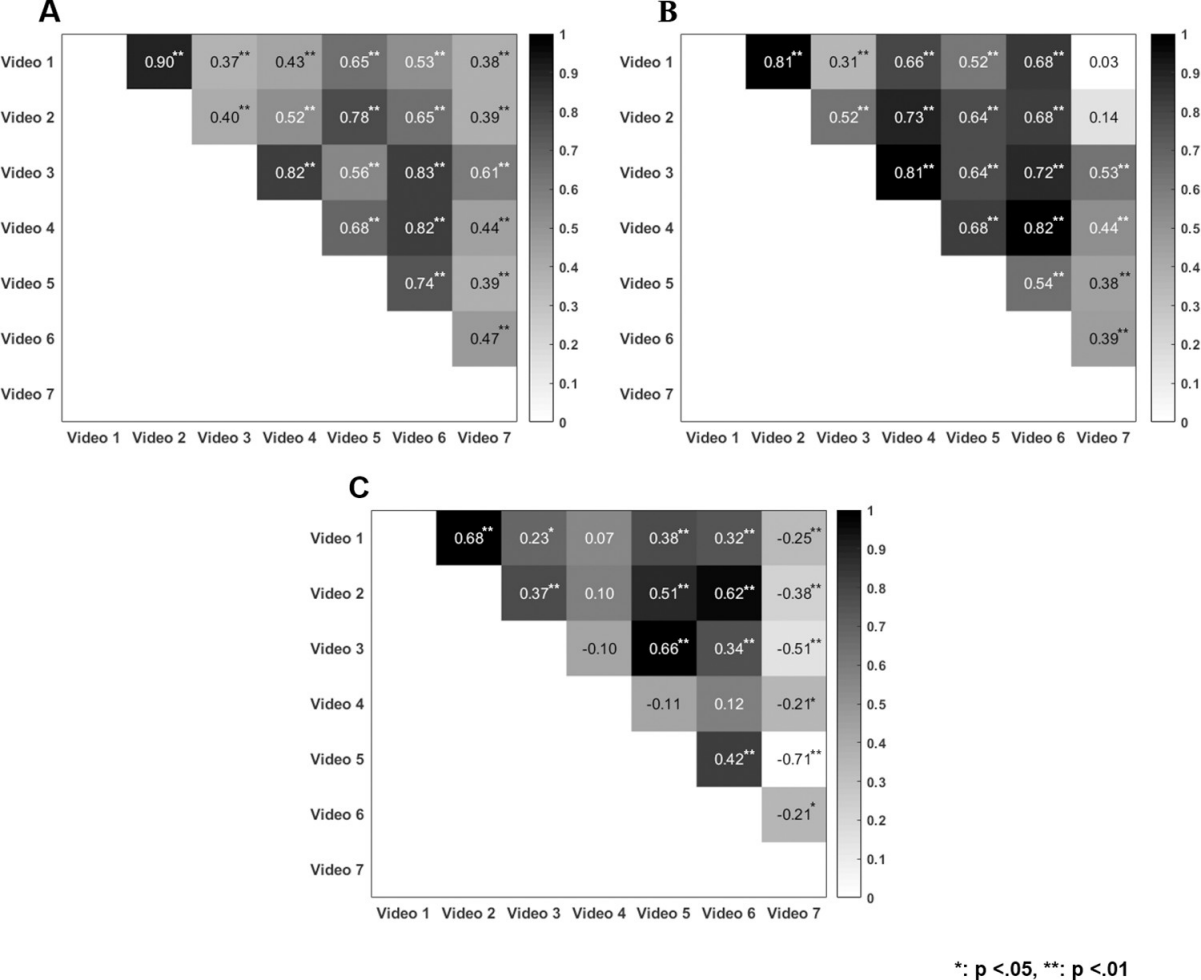
Correlations between the same ratings across different videos for (A) actual speed, (B) subjective speed, and (C) subjective risk.

Second, we found that the time-resolved correlations of the same ratings (see Fig 2) for actual speed were significant for all videos (all p < .001). Similarly, except for correlations of video 7 with videos 1 and 2, all correlations for subjective speed were significant, which indicates that all cars were driving at a similar speed. Correlations for subjective risk were significant except for those of video 4 with videos 1, 2, 5, and 6; however, the overall correlation values lower than those for subjective speed, which indicates differences in subjectively perceived risk between the different videos.

**Fig 3:**
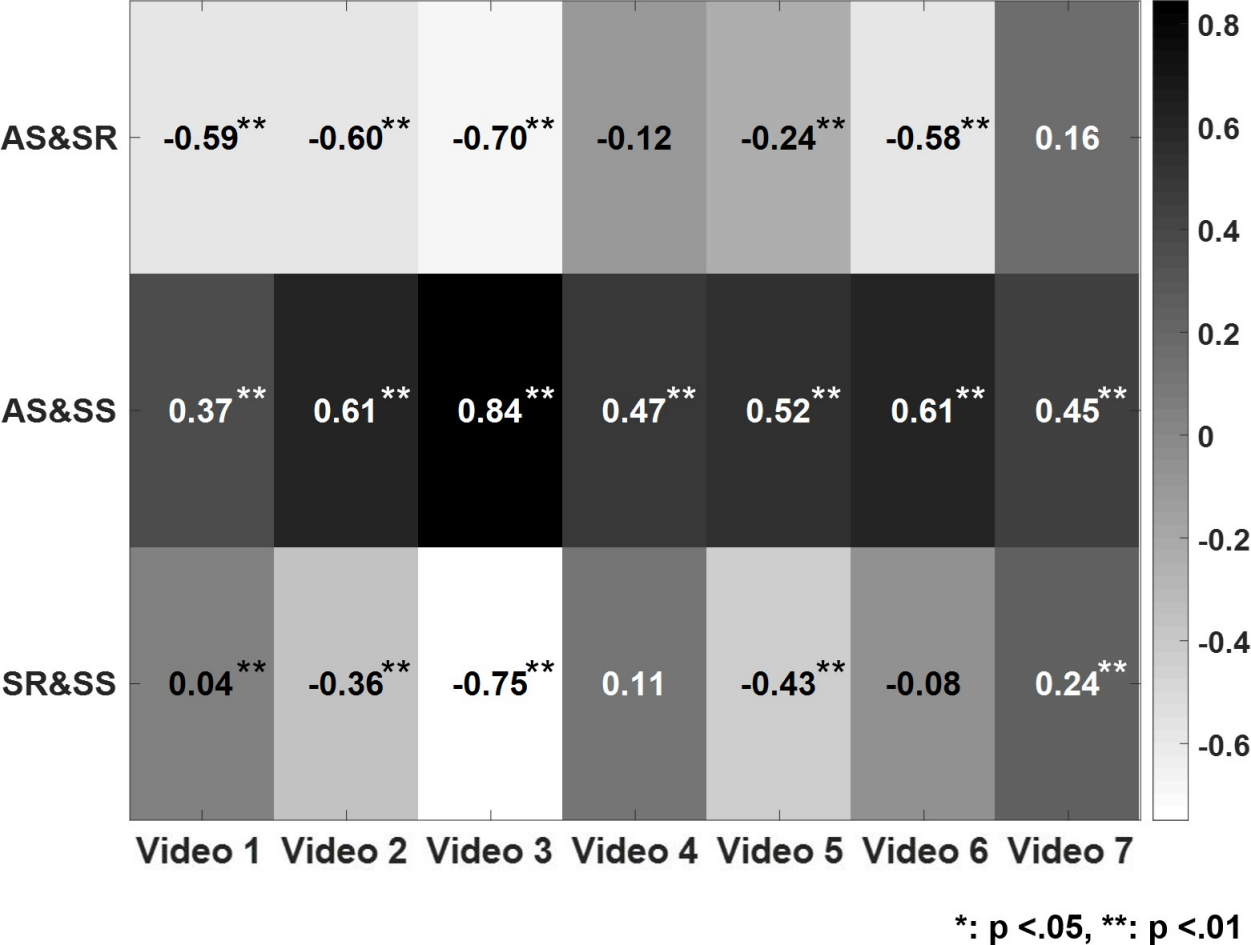
Correlations between different ratings across different videos. AS: actual speed, SR: subjective risk, SS: subjective speed.

Third, our analyses revealed positive associations between actual and subjective speeds across all videos (all p < .001; see Fig 3), except for videos 4 and 7, for which actual speed and subjective risk were negatively correlated. Additionally, subjective speed and risk were negatively correlated for videos 2, 3, and 5 (all p < .001) and positively correlated for video 7 (p < .009).

### Dynamic rating analysis of risk and speed

**Fig 4:**
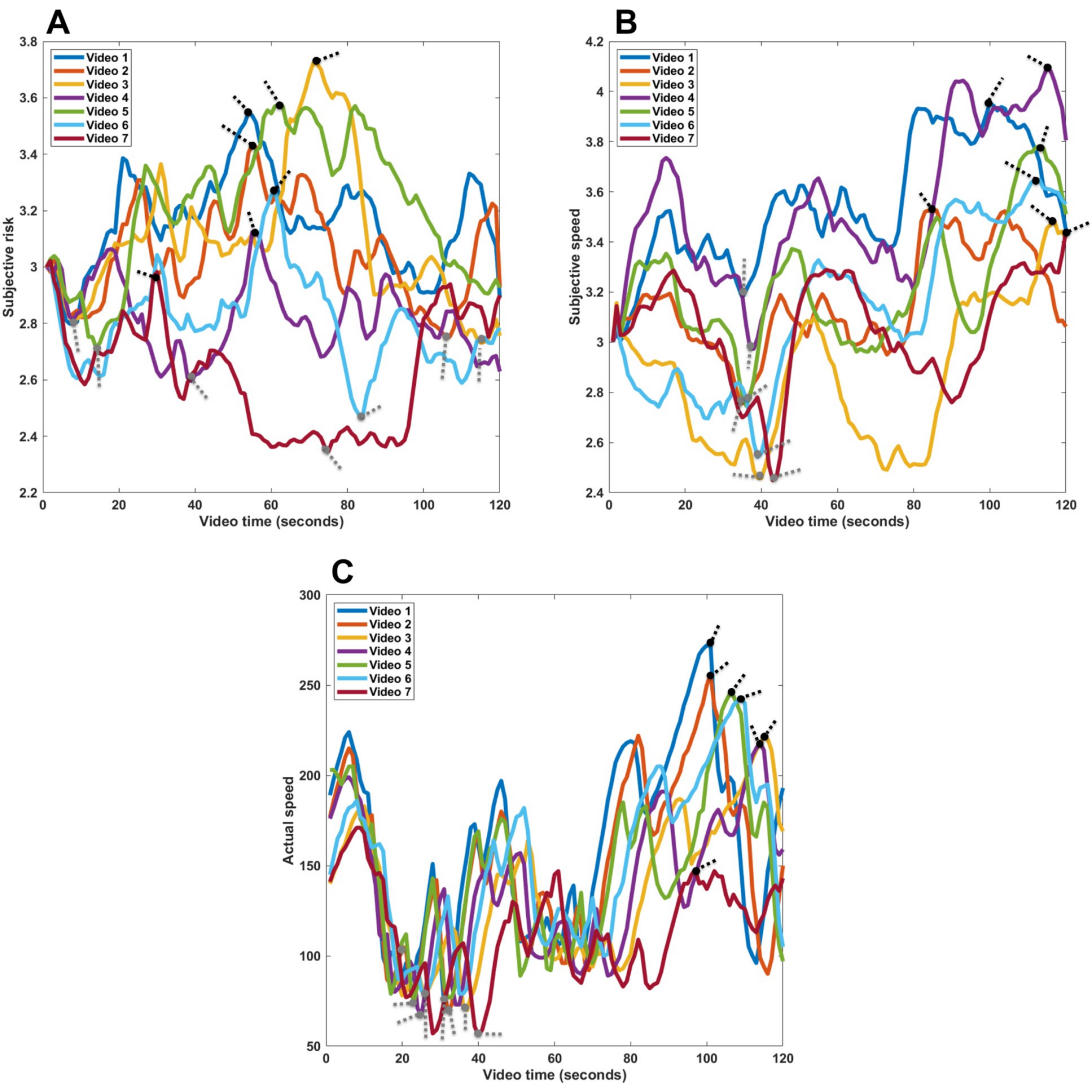
Dynamic ratings for all videos: (A) subjective risk, (B) subjective speed, and (C) actual speed. Black and gray dots with dotted lines indicate maximum and minimum points, respectively.

We extracted the dynamic ratings of subjective risk, subjective speed, and actual speed to investigate the situational factors that influence risk and speed perception. The maximum and minimum risk points were widely distributed, whereas the actual and subjective maximum and minimum speed points were located at similar points along the track (see Fig 4). Our analysis of the footage taken 3 seconds before and 3 seconds after the maximum and minimum risk points revealed that “maximum risk situations” occurred during bends in the track, when other cars were on the road ahead, when the distance to the car in front decreased, or while the car was being overtaken (Table 2). Similarly, “minimum risk situations” occurred mostly during sequences in which the road was straight, no cars were up ahead, or the distance to the car in front increased (see Table 2).

**Table 2:** Situational analysis of all seven videos: Actual speed, subjective speed, and subjective risk derived from the video clip of 3 seconds before to 3 seconds after the maximum and minimum time points.

	Track type (straight/curved)	Car in front (yes/no)	Distance decreases (yes/no)	Matches actual speed (yes/no)
Actual_speed_max	7/0	3/4	0/3	N/A
Actual_speed_min	0/7	4/3	2/2	N/A
Subjective_speed_max	7/0	3/4	3/0	4/3
Subjective_speed_min	0/7	3/4	1/2	3/4
Subjective_risk_max	0/7	7/0	7/0	N/A
Subjective_risk_min	5/2	4/3	0/4	N/A

Our analyses revealed that the subjective maximum speed time points on the straight parts of the track were close to the actual maximum speed points (Fig 4C). In addition, if there was a car in front, the distance to that car decreased at the maximum speed (see Table 3). In contrast, the minimum speed points mostly correlated with bends along the track, and if there was a car in front, the distance to that car increased at those points in time. Overall, our results indicate that the driving track characteristics and the presence of cars in front influenced ratings of risk and speed.

**Table 3:** Driving content of all videos and overall risk and speed evaluations.

	No. of overtaking maneuvers	No. of times the car is overtaken	Distance decreases (seconds)	Distance increases (seconds)	Chosen as the fastest sequence	Chosen as the slowest sequence	Chosen as the most dangerous sequence	Chosen as the safest sequence
Video 1	4	0	28	18	27	2	11	8
Video 2	3	0	22	98	4	15	5	8
Video 3	2	0	19	101	2	24	22	0
Video 4	6	0	45	11	35	1	5	17
Video 5	5	0	26	31	3	12	24	6
Video 6	2	1	12	24	6	12	5	15
Video 7	3	1	37	58	1	12	6	24

### Relationship between driving context and risk/speed estimation

Based on our previous analyses showing that driving context influences subjective maximum risk and speed, we next determined the association between driving context and overall speed and risk perception. To that end, we extracted the number of overtaking maneuvers, the number of times the car was overtaken by other vehicles, and the number of times the distance from the car in front increased or decreased (see Table 3). We then applied Spearman’s correlation analysis to investigate associations between driving context and the sequences chosen as the slowest, fastest, most dangerous, and safest. We found that increasing distance to the car in front was negatively correlated with a sequence being chosen as the fastest (r = −0.82, p = .034) and positively correlated with a sequence being chosen as the slowest (r = 0.96, p = .002). Next, we assessed potential relationships of average dynamic risk and speed ratings as well as actual speed with driving context, overall speed ratings, and risk. We found that average dynamic speed ratings were significantly correlated with the number of overtaking maneuvers (r = 0.81, p = .041), an increasing distance to the car in front (r = −0.78, p = .048), a sequence being chosen as the fastest (r = 0.78, p = .048), and a sequence being chosen as the slowest (r = −0.78, p = .045). In contrast, only a sequence being chosen as the safest was significantly correlated with average dynamic risk ratings (r = −0.83, p = .028). No significant correlation was found for actual speed. Overall, we found that subjective speed was significantly associated with driving context, and that dynamic speed and risk ratings were significantly correlated with overall speed and risk evaluations.

### Dynamic rating analysis of over- and underestimations

**Fig 5:**
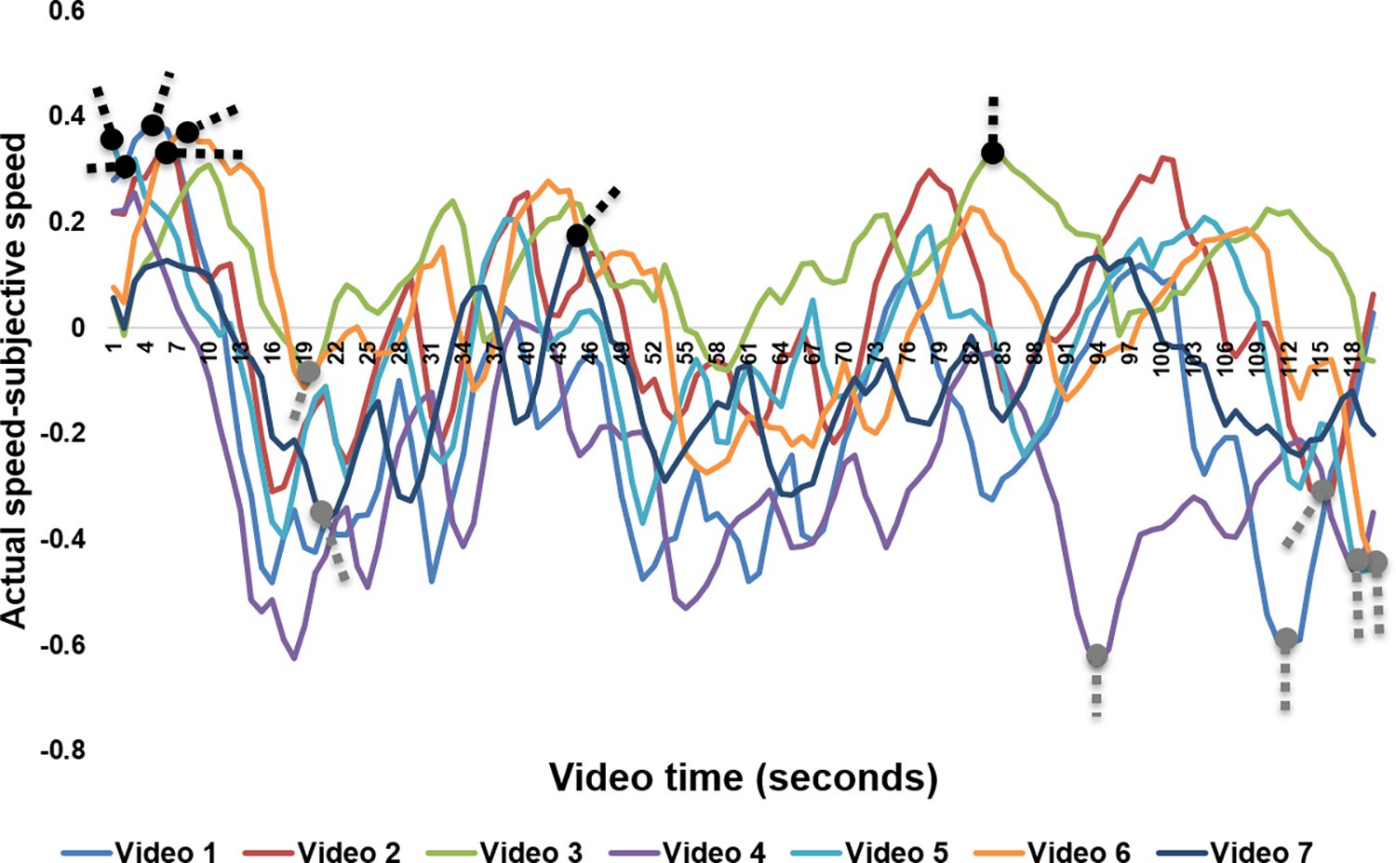
Speed estimation differences for all videos. Black and gray dots with dotted lines indicate maximum overestimations and maximum underestimations, respectively.

**Fig 6:**
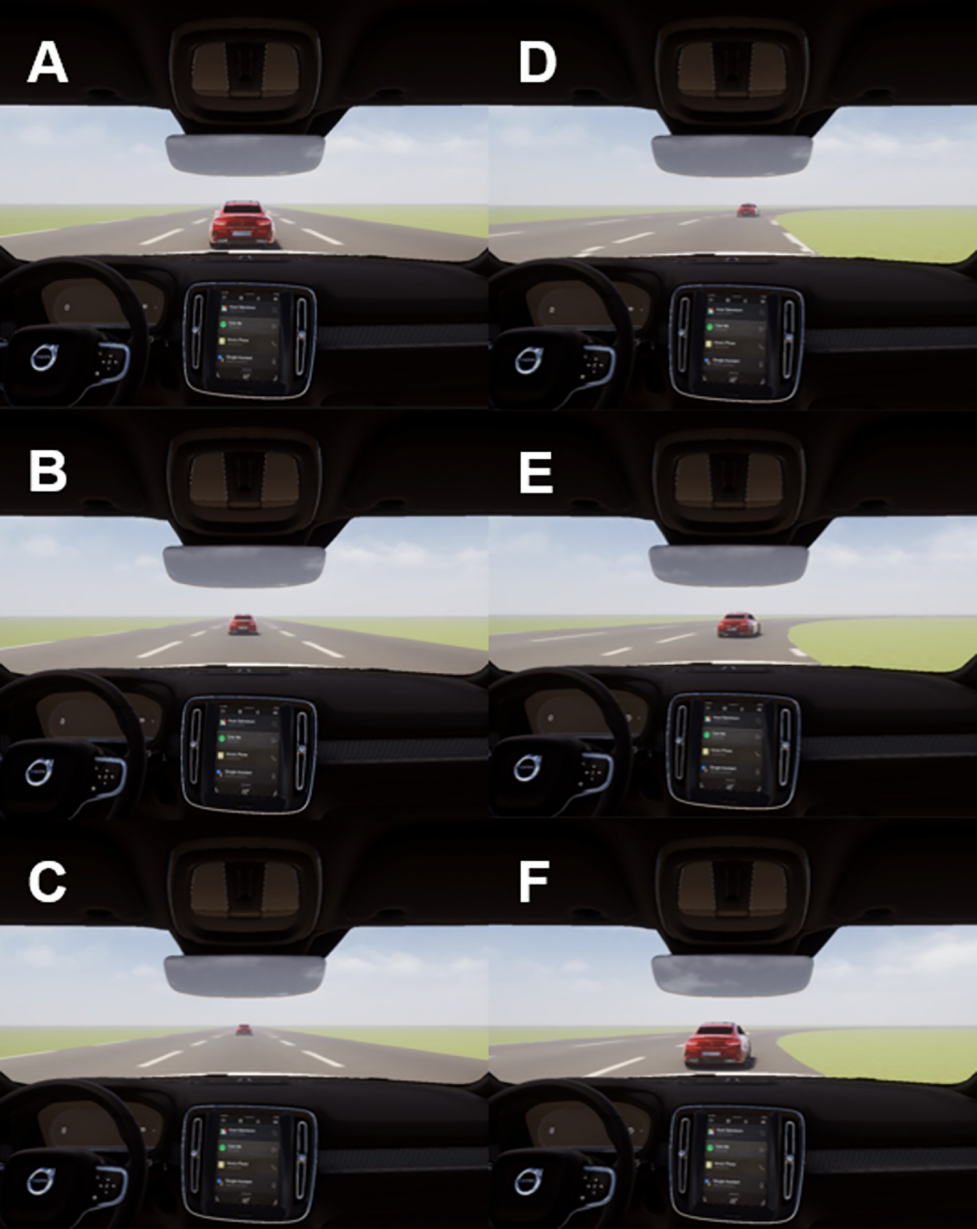
Examples of time points in which maximum underestimations and overestimations occurred (taken from video 3): (A) 3 seconds before the underestimation time point, (B) maximum underestimation time point, (C) 3 seconds after the underestimation time point, (D) 3 seconds before the overestimation time point, (E) maximum overestimation time point, and (F) 3 seconds after the overestimation time point. Due to copyright issues, the figure has been replaced with a self-generated image using Unity 3D that is similar but not identical to the original image used in the study, and is therefore intended for illustrative purposes only.

Since the previous analysis showed that actual driving speeds did not generally correlate with perceived speeds or driving context, we compared actual and subjective speed to determine the time points at which the misestimations with the greatest impact occurred. Our analysis of normalized value differences between actual and subjective speeds across all videos revealed that five out of seven maximum underestimation and maximum overestimation time points were located at similar positions along the driving track (see Fig 5). Moreover, all speed underestimations occurred during driving sequences on straight roads when the vehicle’s speed was increasing, the distance to the car ahead was increasing, or there were no cars ahead at all (see Fig 6A–6C and Table 4). All speed overestimations, in contrast, occurred on curves, when the driving speed was decreasing, when the distance to the car in front was decreasing, or when there were no cars in front (see Fig 6D–6F and Table 4). These findings indicate that track characteristics and changes in distance to the car in front were the main reasons for misestimations.

**Table 4:** Situational characteristics of the time points where maximum under- and overestimations occurred.

	Track type (straight/curved)	Car in front (yes/no)	Distance decreasing (yes/no)	Speed change (increase/decrease)
Maximum underestimation	7/0	3/4	0/3	7/0
Maximum overestimation	0/7	5/2	5/0	0/7

## Discussion

In this study, we employed a dynamic rating method to measure the subjective speed and risk of first-person perspective videos of cars driving on the same race track. To the best of our knowledge, this is the first study to use videos of race car driving on the same racing track to dynamically assess risk and speed perceptions. We found that subjective risk and speed perception were significantly influenced by road characteristics and the distance to the car in front. We also found that speed misestimations could be attributed to a combination of road type, distance to the car in front, and accelerations/decelerations in driving speed.

### Correlations between videos for same behavioral ratings

We found positive correlations for actual and subjective speed across videos for the same behavior ratings and time points, and both positive and negative correlations across videos for subjective risk. These results suggest that driving speeds were similar across the same driving track, regardless of driving skill and performance, whereas risky situations varied and depended on the movements of other cars. In brief, our findings demonstrate the usefulness and reliability of our dynamic approach for identifying the factors that influence perceived risk and speed.

### Correlations across different behavioral ratings

Our first hypothesis was that subjective speed would significantly correlate with actual speed. We did indeed find such a significant correlation for all videos, reflecting that participants’ dynamic ratings were moderately accurate in reflecting dynamic changes in driving speed. Additionally, we found that subjective risk was negatively correlated with both actual and subjective speed. These findings imply that perceived low speed during driving is associated with high risk, potentially influenced by factors such as curves in the road or the presence of a vehicle ahead.

### Characteristics of maximum/minimum speed and risk points

Our second hypothesis was that the time points at which subjectively perceived speed and risk are at their minimum/maximum are characterized by similar road conditions and situational factors. We found that all maximum risk time points were associated with similar positions along the race track, which was S-shaped, and that there was always a car in front in such situations. In contrast, the minimum risk time points had no road characteristics in common, and the only similarity between them was that there was either no car in front or an adequate distance between the driver’s car and the one in front. These results show that a perception of high risk was induced by both road characteristics and situational factors in our experiment, which is in line with previous studies reporting that road characteristics [28, 42, 43] and the presence of other vehicles [27] influence perceived risk. In contrast, low risk was only correlated with the presence of other cars, implying that regardless of road characteristics, participants felt safe when no other vehicles were around. Second, our analysis of maximum and minimum speed time points revealed that the perceived maximum speed points were located along the long straights of the race track, while the perceived minimum speed points were located in the curves; this finding is supported by previous research indicating that participants underestimate their speed on curves [11, 12]. Importantly, half of the actual maximum and minimum speed time points matched the subjective maximum and minimum speed points, and the remaining half were located at similar positions along the track. Additionally, if a car was in front of the driver at the maximum speed perception point, the distance to that car decreased around that point in time, whereas at the minimum speed perception points, the distance to the car in front increased, indicating that distance to cars in front is another important factor influencing subjective speed perception. Overall, these findings support our second hypothesis.

### Relationship between situational context and subjective perception

Our third hypothesis was that situational factors and road characteristics are associated with subjective speed and risk. We found that only subjective speed was associated with the number of overtaking maneuvers and the distance maintained from the car in front. These results are supported by those of a previous study, which showed that changes in the distance between one’s own car and cars in front influence subjective speed perception [44]. However, in the present study, subjective risk was not associated with the number of overtaking maneuvers and the distance maintained from the car in front. A potential explanation for this observation may be that perceived risk only changes when a sudden event occurs or the situational context changes [45]; an overtaking maneuver or an increase in distance to the car in front might not be sufficient to induce a significant change in risk perception. These findings partially support our third hypothesis, in that situational context was significantly correlated with subjective speed in our experiment.

### Characteristics of speed misestimations

Our fourth hypothesis was that our correlational analysis of actual and estimated speeds would allow us to identify driving situations that lead to over- or underestimations of speed. The differences between actual and subjective speeds that we observed indicate that speed overestimations occurred during curves when the driver was overtaking or chasing other cars, whereas underestimations occurred on the straights when the distance to the car in front increased. These observations can be explained by the theory of relative velocity, which postulates that speed perception is based on relative rather than absolute speed [46]. For example, the data displayed in Fig 6A–6C suggests that participants may have assumed that the distance to the car in front was increasing due to their own slow driving speed. In contrast, Fig 6D and 6E suggest that participants may have attributed a decrease in distance to the car in front to their own fast driving speed. These findings indicate that situational differences cause people to misjudge their own speed, and that they cannot accurately judge the absolute speed of their car. Overall, we found that, in line with our last hypothesis, our dynamic rating method allowed us to characterize driving situations that can lead to misestimations of driving speed.

### Limitations of the study

This study has several limitations. First, we used video recordings to measure dynamic risk and speed, and our findings may thus need to be validated in real-world driving situations. However, it is difficult to dynamically measure speed and risk perception during real-world driving, because driving on actual roads cannot be replicated while driving conditions are manipulated; therefore, our approach provides an alternative method for dynamically assessing perceptions during driving. Our approach can be used with state-of-the-art technology to dynamically assess participants’ dynamic perceptions—for example, participants sitting in the passenger seat of a car and wearing augmented reality glasses to enable them to share the driver’s visual perspective. In this instance, the participants are able to dynamically rate perceived risk and speed from the same outlook as the driver. This concept is shared with driving simulators [47]. Another available methodology uses an autonomous driving mode in a real-world or virtual reality scenario, allowing participants to dynamically rate perceived risk or speed from the perspective of the driver.

The second limitation of our study is that all videos were recorded at an average speed of >100 km/h, which may restrict the applicability of our results to real-world driving. However, most highways have a speed limit of >100 km/h, and since fatal accidents are related to maximum speed [48] and faster driving speed is associated with a higher crash rate [49, 50], our results provide important insights into speed and risk perception errors and speeding violations that commonly occur on highways. Future studies may need to apply the same methods to driving slower than 100 km/h to generalize the current results.

Third, our sample did not include older adults; thus, generalizing our results to drivers of all ages may be difficult. However, we did not find significant correlations between age and subjective ratings. Since the effects of age on speed and risk perception have mostly been reported for drivers under 25 years of age [19–21], our findings can be used as a baseline for modeling over- and underestimations in young adults, the age group associated with the highest number of road accidents. Future studies should recruit older adults and assess potential differences in dynamic perception across age groups.

Finally, given that a racing track was used to assess perceived risk and speed in this study, the results may be specific to such environments and thus, their generalizability to real-world situations may be questionable. Therefore, future studies should evaluate perceived risk and speed in various real-world settings, such as urban streets, freeways, and rural roads, to validate and extend the applicability of our findings to more general cases.

## Conclusions

In this study, we used a dynamic rating method to investigate continuous changes in speed and risk perception according to situational factors along a racing track. Our findings indicate that road characteristics and changes in distance to the car in front influence subjective speed and risk perceptions, and that the distance to the car in front correlates with over- and underestimations of speed. Our results provide important insights and an alternative method for measuring dynamic changes during naturalistic driving. This method has advantages over the commonly used verbal reports or post-experiment ratings and can help to identify various factors that lead to misestimations of speed and risk; such insights will be especially relevant to enhance the safety of intelligent driving assistant systems. For instance, future camera monitoring systems may be able to detect driving situations that induce misestimations of speed and risk and warn drivers or provide them with accurate information to prevent problems and ensure safe driving.
